# Prevention of Food Waste in China: Role and Impact of China’s Anti-Food Waste Law

**DOI:** 10.3390/foods13233940

**Published:** 2024-12-06

**Authors:** Shenghang Wang, Dongxu Liu, Jiping Sheng

**Affiliations:** 1 Data Application and Data Governance Innovation Team for the Implementation of Strategy on Comprehensive Law-Based Governance, School of Law, Shandong University, Qingdao 266237, China; ldx_law@mail.sdu.edu.cn; 2 School of Agricultural Economics and Rural Development, Renmin University of China, Beijing 100872, China

**Keywords:** food waste, food sustainability, anti-food waste law, accountability mechanisms, multi-dimensional co-regulation

## Abstract

In recent years, despite global improvements in development, food scarcity and waste remain critical issues impacting food security, human health, and environmental sustainability. China’s Ministry of Agriculture and Rural Affairs reports that China’s food loss and waste rate is approximately 22.7%, amounting to 460 million tons annually, with the consumption and post-harvest processing stages facing the highest losses. To address these issues, China enacted the Anti-Food Waste Law in 2021, aiming to reduce food waste through clear responsibilities, incentives, and penalties for government agencies and relevant stakeholders. While scholars note that the law represents a significant shift from moral to legal governance in tackling food waste, some argue that its provisions lack sufficient specificity. This article assesses the effectiveness of China’s anti-food waste legislation using case studies and comparative analyses, highlighting the challenges in defining and implementing the law within China’s cultural framework, where food signifies abundance and prosperity. Additionally, the article explores successful international practices, including the 2030 Champions Program and similar efforts in Europe and Japan, to inform China’s strategy. The key recommendations for policy improvement include strengthening accountability and governance, establishing a food hierarchy for sustainable resource management, enhancing data collection on food waste, promoting food donation mechanisms, and transitioning from a solely government-led regulatory approach to a multi-dimensional co-regulation model.

## 1. Introduction

In recent years, although global development levels have continuously improved, food scarcity and food waste remain a focal issue in the global media and public attention regarding food security, human health, and environmental sustainability. According to data from China’s Ministry of Agriculture and Rural Affairs, China’s food loss and waste rate is approximately 22.7%, resulting in a total loss of about 460 million tons of food. The two stages with the most significant losses are consumption and post-harvest processing, which account for 158 million tons and 157 million tons, respectively. From a nutritional perspective, the amount of food lost and wasted could meet the nutritional needs of 190 million people for one year. Economically, the losses caused by food waste are as high as 1.88 trillion yuan, equivalent to 22.3% of the agricultural output value [1]. Globally, in an assessment of the level of food waste in the EU28, Stenmark et al. (2016) report that households and food service entities generate 57 million tons of food waste, which represents 65% of the total food waste in the food chain [2]. In 2021, the United States produced 91 million tons of surplus food, or about 38% of the total food supply. This equates to an average of 548 pounds of extra food per person, valued at $444 billion, or about 2% of the gross domestic product of the United States (ReFED, 2022) [3]. The 2024 UNEP Food Waste Index Report states that in 2022, humans generated 1.05 billion tons of food waste, which is close to one-fifth of the food available to consumers [4].

Indeed, each individual country plays a pivotal role in addressing the intricate challenges surrounding food shortages and food waste. On 29 April 2021, the Anti-Food Waste Law of the People’s Republic of China was enacted. This law outlines the principles and requirements for combating food waste, delineates the responsibilities of the government and its agencies, specifies the obligations of the various stakeholders, and establishes both incentives and penalties, as well as legal consequences for non-compliance. The Chinese government has issued a series of administrative regulations and policies aimed at preventing food waste, ensuring national food security, and promoting sustainable economic and social development.

Some scholars believe that the implementation of this law has facilitated a historic transformation in China’s longstanding fight against food waste, shifting from moral constraints to a framework of legal governance [5]. However, there is a perspective suggesting that the provisions of the law lack sufficient specificity, functioning primarily as a declaration of values, which may hinder its effective implementation. This article seeks to analyze the effectiveness and role of China’s anti-food waste legislation through the utilization of case studies and comparative analyses.

The assertion that the law lacks specificity warrants closer examination; while it may function as a broad statement of values, its effective implementation hinges on the subsequent regulations, guidelines, and public awareness campaigns that elucidate its provisions. The success of these accompanying measures will ultimately determine the extent to which the law translates into tangible changes in behavior and practices. To assess the legislation’s effectiveness, case studies provide valuable insights. By examining specific instances where the law has been invoked, we can evaluate its practical impact on reducing food waste while also highlighting the challenges encountered during the implementation. Furthermore, the cultural context surrounding food waste in China—where food often symbolizes abundance and prosperity—poses a significant challenge. Defining food waste within this cultural framework is crucial, as changing entrenched attitudes and behaviors requires not only legal sanctions but also sustained efforts to educate and influence public opinion.

Addressing food waste effectively requires not only individual initiatives to seek solutions but also an examination of the successful experiences and practices of other countries. Through a comparative analysis of similar legislation in various nations, we can glean valuable lessons about combating food waste in China. Firstly, it is essential to develop a comprehensive understanding of how other countries address food waste at the legislative level. Notable examples include the 2030 Champions Program, relevant food waste legislation in Europe, and similar initiatives in Japan, which shares a comparable cultural context. Secondly, we must analyze the legal mechanisms and models employed by these countries and regions to tackle food waste effectively. However, it is crucial to recognize that the national conditions and specific contexts of each country differ significantly. While we can draw lessons from international experiences, we must also innovate and adapt strategies that align with China’s unique circumstances. This approach will ensure that our anti-food waste efforts are both relevant and effective in promoting sustainable practices.

Based on the above background, the structure and main content of the article include, firstly, “China’s Anti-Food Waste Law: Key Provisions and Implementation”, which will examine the fundamental provisions of the law, including the definition of food waste and the measures designed to mitigate its occurrence. This section will also explore the practical application of the law through illustrative case studies. Subsequently, it will present a “Comparative Analysis: China’s Anti-Food Waste Law and Global Practices”, which will evaluate the legal frameworks and case studies related to anti-food waste legislation in the United States, the European Union, and Japan, assessing the potential implications of these approaches for China. Finally, it will provide “Recommendations for China’s Policy Improvement”, identifying critical issues for the future governance of the Anti-Food Waste Law, including improving accountability mechanisms and building a comprehensive governance framework; establishing a systematic operational mechanism to address food waste across the entire chain; developing a food hierarchy system to promote sustainable resource management; establishing a food waste measurement mechanism to enhance data collection capabilities; improving food donation mechanisms to prevent waste at the end of the supply chain; and shifting the regulatory model from single government regulation to multi-dimensional co-regulation.

## 2. China’s Anti-Food Waste Law: Key Provisions and Implementation

The enactment of China’s Anti-Food Waste Law is based on the broader context of global food waste, the promotion of food waste legislation in various countries, and food security. Research by the Food and Agriculture Organization of the United Nations (FAO) shows that globally, one-third of food and the resources it carries are wasted every year, a figure that is as high as 1.3 billion tons [6]. According to the quantitative investigation by Ling-en Wang (2017) of food waste in restaurants in Beijing, Shanghai, Chengdu, and Lhasa, per capita food waste in these four cities was 93 g per meal, consisting mainly of vegetables (29%), rice (14%), aquatic products (11%), wheat (10%), and pork (8%) [7]. The seriousness of food waste means the main provisions and implementation process revolve solely around the purpose and core of practicing economy and combating waste.

### 2.1. Overview of Key Provisions

#### 2.1.1. Legal Obligations for Individuals, Businesses, and Institutions to Reduce Food Waste

China’s Anti-Food Waste Law is a mixture of top-down and bottom-up models. On the one hand, the government plays a central role in policy formulation and implementation, and other stakeholders implement or participate in anti-food waste actions under the guidance of the government (Han Huang et al., 2023) [8]. On the other hand, the media play a role in disseminating and guiding public opinion in the process of policy formulation and implementation, reflecting matters of wide public concern (Jackson et al., 2021) [9]. This makes it possible to regulate not only governmental organizations and institutions but also the media and the public, among others.

The discussion of the regulations against food waste in China has been going on for a long time. Huang Xisheng et al. (2021) conducted a comparative study of the United States, the European Union, Japan, and other countries and regions, pointing out that China’s food conservation legislation lacks accountability and a comprehensive governance mechanism [10]. Zhao Jiechun et al. (2022) analyzed the laws and regulations to reduce food waste in Japan, and proposed to build a system that emphasizes the role of the state, local public organizations, enterprises, and consumers [11]. Zhang Yaoyao et al. (2023) analyzed the current situation of food waste and governance dilemmas in China and put forward the following suggestions: strengthening the horizontal and vertical collaboration of functional departments and shaping the pattern of multi-constituent co-governance [12]. Lok Chi-kwan (2021) believes that the core of the anti-food waste legislation should take the theory of the sociality of resources as its jurisprudential basis, and should require the fulfillment of the requirements of social and environmental obligations of property rights [13]. Seed planning (2022) analyzed the current situation of food waste reduction in the United States, Australia, Germany, and China, and proposed that the regulation of catering operators should be strengthened and consumer education should be enhanced [14].

China’s Anti-Food Waste Law has a total of 32 articles and clearly stipulates the duties and obligations of government departments and other subjects. With regard to the departments of the State Council, it is stipulated that the Development and Reform Department of the State Council, as the main responsible department, is responsible for coordinating the national anti-food waste work and the overall deployment of the anti-food waste work. Among them, the Competent Commercial Department, the Market Supervision and Administration Department, and other relevant departments carry out corresponding anti-food waste work within their respective areas of competence.

The obligations of different subjects are regulated in different ways. First, the legislation requires food service operators to establish and improve food management systems and business practices in a targeted manner, according to their own business characteristics. Secondly, food takeaway platforms are required to prompt consumers to order reasonable and appropriate quantities of food in a conspicuous manner. Third, other food operators are required to strengthen the daily inspection of the food they operate, categorize and manage food approaching its shelf life, and make special labels or centralized displays for sale.

China’s Anti-Food Waste Law has realized the joint governance of the State, public organizations, enterprises, and consumers, forming a “circular” legislative system and moving toward a dual green development legal system of green consumption law and green production law. At the same time, the popularization of consumer education and the concept of green development have become new legal elements.

Of course, there are many problems in the legislation. China’s Anti-Food Waste Law mainly focuses on regulating the consumption stage at the end of the supply chain. However, the regulation of the front-end link of the supply chain at the processing stage is relatively void, being limited to Article 15 of the Anti-Food Waste Law, which requires that the State and food production operators should take measures to reduce food loss, and Article 28, paragraph 3, which stipulates that food production operators may be ordered to make corrections or be fined if they have caused serious food waste in the course of their food production and operation, which is extremely imbalanced when compared with the rich content of the consumption stage of the governance.

#### 2.1.2. Penalties for Excessive Food Waste in Restaurants, Events, and Public Institutions

The Anti-Food Waste Act adopts a categorized approach to governance, utilizing the legislative idea of combining constraints and advocacy, and, based on the differences in the intrinsic driving force of the different subjects in reducing food waste, introduces corresponding regulatory measures according to the categorization of the subjects, thus providing a basis for law enforcement agencies to stop food waste. The four main categories are food service operators, food production operators, units with canteens, and radio and television stations, as well as network audio and video service providers. If a food service operator fails to take the initiative to remind consumers of food waste prevention tips, the relevant department can order correction and give a warning; if it induces or misleads consumers to over-order, resulting in obvious waste, the relevant department can order correction and give a warning or even a fine. If a food production operator causes serious food waste in the process of food production and operation, the relevant department may order correction or impose a fine. If a unit with a cafeteria fails to formulate or implement measures to prevent food waste, the relevant department may order correction and give a warning.

The penalty mechanism of China’s Anti-Food Waste Law clearly stipulates the responsibilities of the various types of subjects in food production and consumption, and creates a multi-level penalty ranging from warnings to fines to suspension and reorganization, which not only embodies the seriousness of the law but also enhances the flexibility and operability of the law.

However, the lack of detail in the content related to the administrative penalties increases the difficulty of law enforcement officers in enforcing the law and leads to a monolithic approach to law enforcement, which affects the stability and predictability of administrative law enforcement. First, there are too few subjects of regulation. Although Articles 28 to 30 of the Anti-Food Waste Law stipulate the areas in which food waste is the most prominent problem, it does not stipulate whether or not there is administrative punishment for other wasteful behaviors, such as the failure of network service platforms to fulfill their obligations and management responsibilities, or the seriousness of individuals wasting food. Second, there are too few types of administrative penalties. According to the statistics of administrative law enforcement cases, the administrative law enforcement departments are mainly punishing for two types of violations: “failure to take the initiative to remind consumers to prevent food waste tips” and “inducing, misleading consumers to order more than the amount of food”. Third, there are fewer types of administrative penalties. On the one hand, there are only three types of administrative penalties, namely, “ordering correction, warning and fine”, which do not involve qualification penalties and behavioral penalties, making it difficult for them to play a sufficiently educative and cautionary role. On the other hand, given the complexity of social life, the small number of types of penalties makes it difficult to meet the needs of real-life problems, which in turn affects the smooth operation of the administrative law enforcement agencies.

### 2.2. Defining Food Waste and the Causes of Food Waste

In September 2015, the United Nations (UN) General Assembly adopted 17 Sustainable Development Goals (SDGs), including “responsible consumption and production” and “zero hunger”. According to relevant reports, reducing food waste at the consumer end and shifting dietary choices to a balanced, sustainable, and healthy diet is important for promoting sustainable development (IPCC, 2022). [15] Shreya Some et al. (2022) identified food waste reduction as a low-demand mitigation option to achieve the SDGs [16]. Anais Lemaire (2019) found that reducing food waste can be a sustainable consumption and production pattern [17].

There is no standardized definition of food waste, and different terminologies and methodologies exist (Marija Jeremic et al., 2024) [18]. In general, definitions of food waste often differ in four main elements: supply chain stage, final destination, edibility, and food quality (Chauhan et al., 2021; Spang et al., 2019) [19,20]. The USDA defines food waste as part of food loss (Buzby et al., 2014) [21], while Fusion (2014) equates the two terms regardless of which part of the supply chain they originate from [22]. Parfitt et al. (2010) consider “food waste” to be a decrease in the amount of food at the retail and consumer end of the food supply chain caused by subjective factors related to retailers and consumers [23]. Gustavsson (2011) suggests that food waste starts at the beginning of agricultural production and ends at the end of household consumption, encompassing the entire food supply chain [6]. HLPE (2014) proposes that food waste is food for human consumption that is discarded at the point of consumption [24]. The term food waste in this paper takes a broad interpretation with reference to the legislative intent of China’s Anti-Food Waste Law, i.e., improper consumption of the food available for consumption throughout the entire food chain, including the supply side and the consumption side.

**Figure 1:**
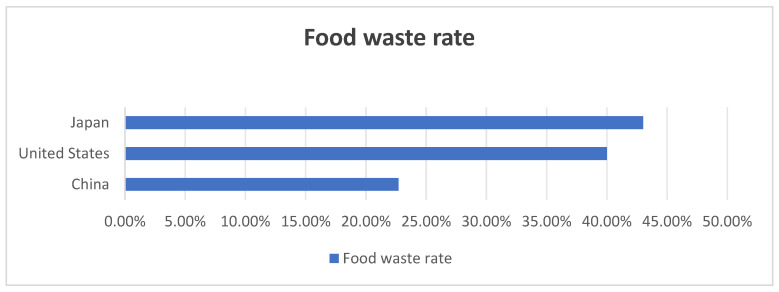
Food waste rates in China, the United States, and Japan.

Data shows that China’s food loss and waste rate is 22.7%, US food waste accounts for about 40% of the entire US food supply, and Japan’s unopened food discarded in its original packaging, the amount of direct waste, and discarded leftovers accounted for 43% of the total (as shown in Figure 1) [1,25,26].

The main reasons for the high level of food waste in rural areas include low economic levels, the influence of traditional practices, poor storage and processing technologies, a lack of knowledge about food management, and inadequate policy and market support. Low economic levels lead to limited income among farmers, who buy or prepare food in excess to show their enthusiasm, which ultimately leads to wastage. Traditional practices, such as weddings, funerals, and festivals, often result in the preparation of large quantities of food in excess of the actual needs. Outdated storage and processing technologies and a lack of proper refrigeration and preservation equipment make food susceptible to spoilage. Residents lack knowledge in food management and do not know how to rationalize food procurement and storage, which accelerates food spoilage. Low levels of education mean that residents do not have sufficient knowledge of the hazards of food waste. Insufficient policy and market support leads to a lack of effective policy measures and market channels, resulting in over-produced food that is not able to be sold or disposed of in a timely manner. These factors combine to exacerbate the problem of food waste in rural areas.

### 2.3. Multi-Stakeholder Participation in Food Waste Management

The implementation of government policies often has the effect of guiding the public’s willingness to participate in an activity and their planned behavior, and can enhance their intentions to reduce food waste, while perceptions of strong government control can be counterproductive (Boqiang Lin et al., 2021) [27]. The theory of the planned behavior model has been used to understand a variety of environmentally beneficial behaviors such as waste recycling (Echegaray and Hansstein, 2017) [28], water conservation (Trumbo et al., 2001) [29], and green purchasing behaviors (Yadav and Pathak, 2017) [30]. The model is also the main theoretical framework used to explain food waste reduction (Liao et al., 2018) [31]. The more strongly a person feels the intention to perform a behavior, the more likely the person is to perform the behavior (Ajzen, 1991) [32]. Also, according to the Perceived Consumer Theory, the more control a consumer has over food waste, the higher his or her willingness to perform the behavior to reduce food waste (Stefan et al., 2013) [33]. Therefore, the early implementation of policies with clear guidelines by government agencies on the process of enforcement would enable the public to perceive the purpose and requirements of food waste reduction and be better integrated into the process of action. At the same time, it is not advisable for government agencies to exert too much control over the public in their enforcement policies, as this may lead to public resistance.

The most common reasons for food waste in restaurant services include improper storage, high levels of residue, leftovers, difficulty in predicting customer numbers, forgotten and spoiled food, high economic and environmental costs, and difficulty in meeting diversified consumer needs (Ofei and Mikkelsen, 2011) [34]. Thus, it is important for restaurants to offer portion choices to customers and redistribute leftovers to minimize food waste (Leo Sakaguchi et al., 2018) [35]. Article 7 of China’s Anti-Food Waste Law clearly stipulates the obligations of food service operators in terms of management systems and instructions on cueing, the selection of meal specifications, and menu design. Government agencies should strengthen the regulation of food waste in restaurants, with penalties for excessive food waste and incentives for good management.

Food waste in school cafeterias occurs primarily as a result of wastage during preparation and cooking, discarding due to the over-preparation of food, the expiration of the use or opening date, spoilage, and plate waste (Betz et al., 2015) [36]. In order to reduce school food waste, Mirosa et al. (2016) proposed the consumer insight theory, whereby interventions should be made by appealing to students’ values [37]. The educational lunch approach has also become an important way to reduce food waste (Engström Carlsson Kanyama, 2004) [38]. Schools should promote the concept of food conservation through enhanced student education and develop appropriate monitoring and management systems for the structure and number of dining staff.

### 2.4. Case Studies: Implementation of the Anti-Food Waste Law

**Figure 2:**
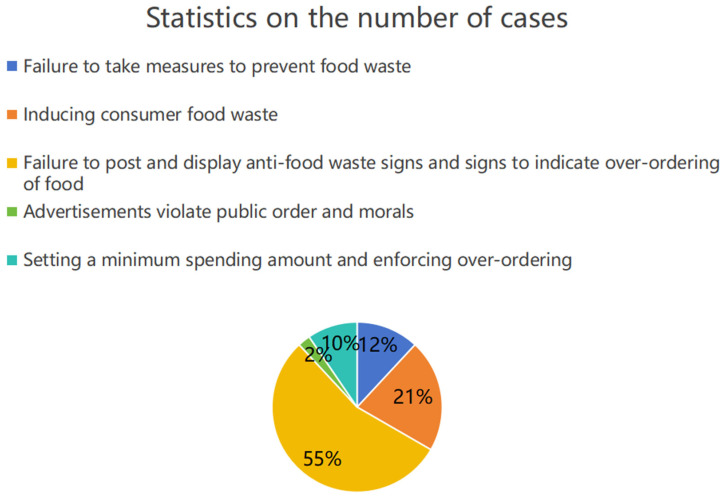
Proportion of different numbers of cases against food waste. (Note: Data from typical cases published by the General Administration of Market Supervision and Administration).

**Figure 3:**
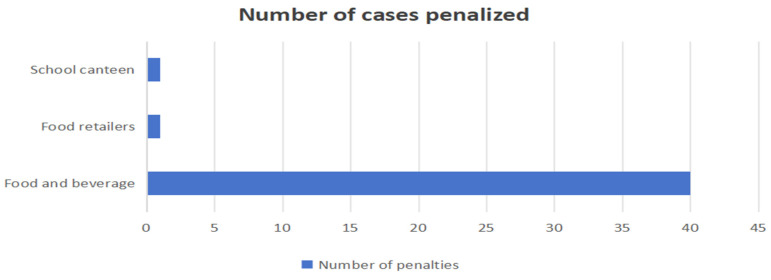
Chart of the number of penalized persons. (Note: The horizontal coordinate indicates the number of penalized cases, and the vertical coordinate indicates the type of market entity that was penalized. The data statistics are for the whole country, from October 2022 to October 2024).

Typical cases show that the administrative penalties are mainly focused on food service operators, with fewer regulations on multiple subjects such as catering platforms, media, schools, and food producers (as shown in Figure 2), which may lead to food waste at the production end and in the transmission chain. In terms of cases, there were 40 cases of caterers being penalized, one of a food retailer and one of a school canteen (as shown in Figure 3).

Shanghai Little Shell Catering Management Co. Ltd. (Shanghai, China) was penalized for publishing advertisements that violated public order and morals. The company published videos and billboards containing advertisements such as “Challenge the whole family to eat the beef bowl within half an hour to enjoy a free order for the beef bowl worth 98 RMB” on online media platforms, and a large number of people took part in the challenge, which resulted in a large amount of waste. The food operator violated its duty to remind people to prevent food waste, and instead encouraged food waste, contrary to the core purpose of the Anti-Food Waste Law. In the case of Yinlong Farm in Pingchao Township, which induced and misled consumers to over-order and refused to correct the situation, the Market Supervision Administration, which had already reminded the farm, knew that it was still violating the law and, for its own selfish purposes, was inducing or misleading customers to over-order when receiving them for a meal, resulting in food wastage, which was contrary to social morality and the provisions of the law.

## 3. Comparative Analysis: China’s Anti-Food Waste Law and Global Practices

In order to prevent food waste, many countries have legislated against food waste with some success [5]. Since the implementation of France’s 2016 Anti-Food Waste Law, the state has empowered thousands of associations and a number of start-ups specializing in the management of unsold food to work with distributors to organize the recovery and redistribution of food, and the total amount of food recovered has increased by nearly 28% per year, which has had a positive impact on stopping food waste [39]. Italy passed an Anti-Food Waste Law in August 2016, which aims to reduce food waste at all points in the food supply chain, focusing on the donation and distribution of discarded food and driving down the total value of wasted food to 0.88% of GDP in 2018–2019 [40]. Japan’s implementation of the Food Waste Reduction Promotion Law, which was passed in 2019, resulted in a 300,000 t reduction in food waste in 2019 compared to 2018 [41]. This section attempts to provide lessons for the development of food waste practices by comparing China’s Anti-Food Waste Law with global practices.

### 3.1. Comparison with Global Initiatives

#### 3.1.1. US Food Loss and Waste 2030 Champions Program

In 2015, the EPA developed the Food Recovery System, which proposes to prioritize the reduction of food waste at the source and, together with the USDA, set the goal of cutting food waste in half by 2030 [42]. In order to achieve the 2030 goal, the Food Recovery Act of 2017 attempts to reduce food waste through source reduction, donations, animal feeding, industrial use, and composting [43]; in 2018, the US federal agencies signed a Formal Agreement for Cooperation and Coordination, which is intended to better educate the American public about reducing food loss and waste [44].

The US 2030 Food Loss and Waste Champions Initiative recognizes organizations that have committed to reducing food loss and waste in their own operations by half by 2030 and that regularly report on their progress [45]. Companies and organizations wanting to join the 2030 Food Loss and Waste Champions need to commit to reducing food loss and waste in their own operations and report progress regularly on their websites. Neither the USDA nor the EPA audits the Champions’ food loss and waste estimates, and it is the responsibility of the 2030 Champions to set their own baselines and measure their reductions. Champions have the discretion to calculate the 50% reduction on an absolute or a per customer/consumer basis and can obtain technical assistance from Sustainable Management of Food points of contact to measure and evaluate the positive environmental benefits of food waste reduction.

This program has had important achievements. From 2016 through May 2022, more than 45 representative commercial food stores, restaurants, food processors, food manufacturers, and food service, hospitality, and entertainment businesses have become 2030 Champions [46]. The most representative companies include Ahold Delhaize, Albertsons, Amazon, Aramark, General Mills, and so on.

In 2022, Amazon strengthened its constructing, purchasing, and distribution systems, reduced its food inventory in North America and Europe, and improved its food-discounting technology to sell more suitable products. Amazon donated 82 million meals worldwide, including more than 30 million meals donated by Whole Foods to local food banks and food rescue organizations, as well as donations from Amazon Fresh, Customer Delivery, Amazon Go, and The Kitchen. In 2022, Whole Foods Market implemented an aggressive organic-waste-sorting program at 449 locations, diverting nearly 108,000 tons of food waste from landfills.

The 2030 Champions Program is a voluntary initiative that focuses on cooperation with the private sector, which sets its own goals and reports on its progress, covering all stages of the food supply chain. At the same time, the US government plays a facilitator role in the program, and thus there are no direct penalties but rather incentives through improved reputation and cost savings.

#### 3.1.2. European Food Legislation for Food Waste Reduction

In recent years, the EU’s legislative efforts against food waste have yielded significant results. The 2015 EU Circular Economy Package sets out requirements for the EU Food Waste Measurement Methodology and the legislation on waste, food, and feed [47]. The 2017 EU Food Donation Guidelines aim to promote food donation and facilitate the redistribution of surplus food [48]. The 2018 EU Waste Framework Directive (revised) incorporates food waste into the Directive, advocates for a system of food waste assessment methods, and encourages food donation [49]. The 2019 Key Recommendations of the EU Platform Action on Food Loss and Food Waste aim to establish an EU Platform on Food Loss and Food Waste to accompany the Circular Economy Package [50]. The 2020 EU Farm to Fork Strategy sets out objectives such as “revising EU rules on date marking of food and legalizing food waste” and “big investments in R&D programs to develop new technologies/ways for food saving and recycling” [51].

Typical European countries with legislation on food waste include France and Italy. France’s 2012 Waste Management Act and the related amendments impose an obligation on private companies to recycle organic waste [52]. The world’s first Anti-Food Waste Law (Garrote’s Law), enacted in 2016, defines the waste hierarchy of food waste and sets legal constraints on the process of food manufacturing, buying, and selling and the corresponding subjects [53]. In 2020, the Law on Anti-Waste and Promotion of Circular Economy Development, which simultaneously prohibits all businesses from destroying (landfilling and incinerating) unsold non-food products, in addition to unsold food products, was enacted [52].

Italy enacted Europe’s first national Good Samaritan Law in 2003, which aims to reduce food waste with a donor exemption [54]. The 2016 Law on Donation and Distribution of Food and Medicines for Social Solidarity and Reduction of Waste expands the scope of food donors and introduces tax incentives to promote food donation [55].

Although some European countries do not have a separate law on saving food, they have been making corresponding legislative efforts and actions. For example, in 2019, the German federal government introduced the National Strategy for the Reduction of Food Waste, which puts forward the goals of halving food waste at the retail and consumer levels and reducing food waste in the production and supply chain by 2030 [56]. In 2017, the Norwegian government introduced the Food Waste Reduction Agreement, a binding agreement between the Norwegian government and food industry organizations, which specifies that food producers, manufacturers, wholesalers, retailers, restaurants, households, and government departments must all take responsibility for reducing food waste in order to promote food conservation and combat food waste [57].

There are core trends in European food waste legislation: Firstly, the reduction of food waste is closely linked to the circular economy and sustainable development, focusing on the optimal use of resources and environmental protection. Secondly, they promote the redistribution of surplus food through the implementation of donation incentives and encourage enterprises to transfer unused food to social organizations. In addition, European laws generally establish clear legal constraints and penalties to ensure strict compliance throughout the supply chain, from production to consumption. At the same time, the laws often include systematic target-setting and evaluation mechanisms, such as specific reduction targets to quantify waste reduction. Finally, government–industry cooperation plays an important role in European legislation, with all parties sharing the responsibility for reducing food waste.

#### 3.1.3. Japan’s Approach to Food Waste

The legal development of cost-saving in the Japanese food system has been a long-term process: In the 1990s, there was a move from end-of-pipe control to full supply chain management, most notably in 2000 with the introduction of the Basic Law for the Establishment of a Circular Society [58]. Specialized legislation against food loss and waste was introduced in the early decades of the 21st century: In Japan, the Food Recycling Law was implemented in 2001, requiring food producers to reduce waste emissions [59]. In 2005, the Basic Law on Food Education was enacted, stipulating that all people, including children, must participate in food education to combat food waste at the source [60]. In the last decade, Japan has been conducting a national campaign. In 2012, the Act on the Promotion of Consumer Education was enacted to advocate ethical consumption and to call on consumers to be aware of the social costs hidden in cheapness and convenience [61]. The Food Recycling Law was enacted in 2016 to control waste emissions from food production, distribution, and consumption [62]. The Food Waste Reduction Advancement Act was enacted in 2019, clarifying the government’s responsibility to avoid food waste [63].

Japan’s food waste reduction of 480,000 t from 2012 to 2018 has been significant in stabilizing the domestic food supply [64]. Japan has constructed a system of cost-saving targets by type and by industry: the amount of food loss, the proportion of nationals taking action against food waste, the recycling utilization rate in the food industry, and the amount of food waste generated per unit of product. A system for investigating, monitoring, and accounting for food losses and a certification system for waste recycling have been constructed and have created a national fervor against food waste [58].

Additionally, Japan has taken a number of concrete action measures to reduce food waste. The Japanese government attaches importance to business practices and adjusts the details of policy programs [11]. For example, in 2012, the Ministry of Agriculture, Forestry, and Fisheries of Japan promoted the initiative of replacing “year, month, and day” with “year and month” in accordance with trading habits and through the extension of shelf life by new technologies [65]. This practice is also used in countries such as the United States and the European Union. Secondly, a food flow and food bank platform should be set up, and a sound system of subsidizing the costs of transportation, inventory storage, and delivery and distribution should be developed to promote planned and rational consumption by consumers.

#### 3.1.4. Comparison of Legal Frameworks and Enforcement Mechanisms

By comparing the legal frameworks of the four countries, collaborative governance serves as an underpinning for rule of law-based governance. Collaborative governance can be categorized into horizontal governance, which refers to inter-governmental cross-sectoral cooperation mechanisms, and vertical governance, which emphasizes the effective participation of the entire food industry chain [12].

Three main frameworks are included under the horizontal synergy model: First is the pluralistic joint model represented by the United States. A multi-sectoral coalition led by the EPA, the United States Department of Agriculture (USDA), and the Food and Drug Administration (FDA) constitutes the main regulatory force against food waste in the United States [42]. The second is the monolithic dominant model represented by Germany. Germany has developed a governance model led by the Ministry of Agriculture, with the participation of federal governments and agencies at all levels. The third is the model of broad participation and cooperation represented by China and Japan. China’s Anti-Food Waste Law provides for shared governance across multiple public sectors. In Japan, there are more than 20 government agencies responsible for food waste control, and the division of responsibilities among these agencies is clearly defined in the law.

In terms of vertical synergies, countries are realizing the importance of participation throughout the food chain. The implementation of EU government policies includes, but is not limited to, fisheries, industrial policy and the internal market, general, financial, and institutional affairs, taxation, agriculture, environment, and consumer and health protection [66]. ReFED and the Harvard Food Law and Policy Clinic have divided their module on reducing food waste into prevention, recycling, and recovery (Buzby et al., 2014) [21]. Japan’s means of combating food waste mainly include the construction of a system of cost-saving targets by type and by industry [67], a food loss survey, a monitoring and accounting system [68], setting up a certification system to incentivize food waste recycling [69], and multisectoral approaches to promote national action against food waste, etc. [70]. The 2017 US Food Recovery Act attempts to reduce food waste through source reduction, donations, animal feeding, industrial use, and composting [43]. The USDA ERS takes into account the parameters of each food spoilage, table surplus, and other forms of food loss and waste estimate [71] to derive a loss-corrected food availability dataset based on previous data systems [72].

### 3.2. Analysis of the Potential Implications of These Global Practices for China’s Anti-Food Waste Law

Comparing the legal framework and the specific implementation of anti-food waste legislation in different countries provides important references that are significant for China’s anti-food waste legislation. First is collaborative governance and multi-stakeholder participation. Global practice shows the importance of multi-stakeholder collaboration in combating food waste. China’s anti-food waste process should be made possible by encouraging the participation of multiple actors, such as private enterprises and social organizations, rather than relying solely on punitive measures by the state’s coercive power. Second, food redistribution and donations are emphasized. A food donation mechanism has not yet been formed under China’s legal framework, and the effect of food redistribution can be enhanced through the introduction of this structural incentive initiative.

Thirdly, public awareness should be raised and education and publicity should be strengthened. Although Chinese law clearly provides for consumer education and publicity activities, in practice it is still in a state of “more than enough”, so education should be the core element of long-term law enforcement. Fourth is the integrated use of big data and technology-driven capabilities. China can build technology-led, data-driven solutions. Extraterritorial practices such as food waste recycling, enterprise motivation, food banks, and other initiatives have their own localization, and how to localize transnational experiences is an urgent issue to be solved.

## 4. Recommendations for China’s Policy Improvement

### 4.1. Improving Accountability Mechanisms and Building a Comprehensive Governance Pattern

From the perspective of administrative penalties, diversified administrative penalties should be adopted to improve the stability and predictability of the administrative enforcement. First, the scope of the subject of regulation should be expanded. The scope of administrative penalties cannot be limited to the consumer side of the industry; the amount of food waste that occurs at the front end of the supply chain is similar to the consumption stage (Karin Schanes et al., 2018) [73] Therefore, “food producers and operators” in the Anti-Food Waste Law should be resorted to at the front end of the supply chain, and food operators should be categorized and regulated. On the other hand, enterprises that violate food waste legislation should be punished for their direct and related responsibilities so that the responsibility can be put onto individual management and inaction can be prevented. Secondly, the methods of administrative punishment should be enriched. Directive administrative penalties such as fines do not act as a deterrent. For repeated violations of the law, action needs to be taken in the form of severe laws and heavy penalties, which should be different for different subjects and food waste types to provide for targeted behavior, such as fines, revocation of licenses, and criminal liability for food production enterprises that repeatedly produce and sell products that do not meet food safety standards.

In the case of consumers, given that the essential nature of the consumption of a product is the use of private rights by the owner, penalizing consumers inevitably gives rise to the suspicion of an expansion of public power, in violation of the statutory principle of administrative penalties. Consumer regulation tends to take the form of encouraging the reduction of food waste, and it is difficult to create penal rights for consumers.

### 4.2. Building a Systematic Operational Mechanism to Address Food Waste Along the Whole Chain in an Integrated Manner

#### 4.2.1. Establishing a Food Hierarchy to Promote Sustainable Resource Management

China lacks a prioritized food waste evaluation plan, and due to the limited nature of resources, the equal treatment of different foods can lead to problems such as unequal distribution and inefficient treatment, which calls for the establishment of a systematic food hierarchy that in turn promotes sustainable resource management. Proper waste management is recognized as a necessary prerequisite for sustainable development (UNEP, 2011) [74]. In 2008, the European Commission introduced the Waste Framework Directive, which will be amended in 2023 [75]. The Directive establishes a five-step “waste hierarchy” based on the prioritization of waste management and treatment according to environmental considerations [76].

**Figure 4:**
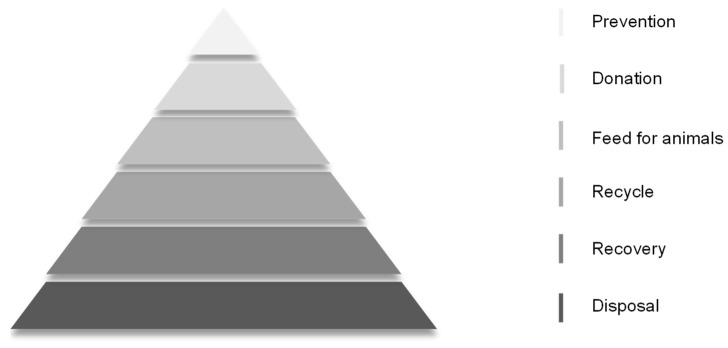
Food handling hierarchy. (Note: According to the EU food disposal hierarchy, the priorities are, in top-down order, prevention, donation, feed for animals, recycle, recovery, and disposal).

**Figure 5:**
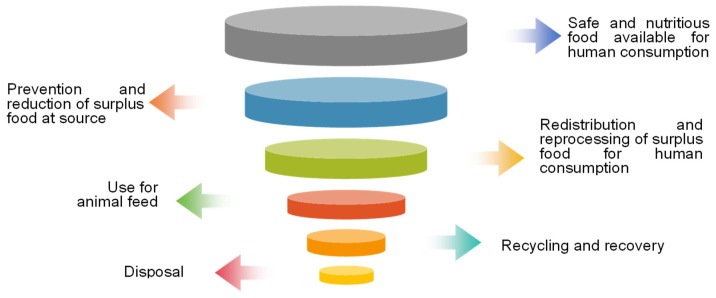
Food use hierarchy.

EU countries have developed food handling hierarchies and food use hierarchies aimed at maximizing the retention of food in the supply chain for human consumption (as shown in Figure 4 and Figure 5) [77].

China can learn from the European Union’s food grading system and use and dispose of its limited resources in gradients by constructing a quality certification system in the areas of food safety, quality certification, regulatory enforcement, transparency and information sharing, and sustainable development.

#### 4.2.2. Establishment of a Food Waste Measurement Mechanism and Upgrading of the Technical Capacity for Data Collection

Another important factor in China’s food waste regulation is that it is difficult to quantify food waste and realize effective monitoring and control. The construction of a food waste measurement mechanism can draw on the institutional systems of niche countries such as the UK and the US. In 2017, the Australian government announced its National Food Waste Strategy, proposing the construction of a food waste data collection system. In 2019, it published the National Food Waste Baseline Assessment Report [78]; in 2020, the 2030 Roadmap to Halve Australia’s Food Waste by 2030 was published by Food Language Australia’s Agricultural Business Development Agency (FIAL) [79]; and it has published a Feasibility Study for a National Food Waste Strategy, and regularly evaluates progress across the country [80]. The FAO database has constructed a dataset of loss rates at different food chain stages for more than 120 food groups in 127 countries worldwide [81].

The United States Department of Agriculture Economic Research Service has the earliest established Food Availability Per Capita Data System [82]. In order to better approximate the real food intake of the population, the Center has taken into account parameters for estimating spoilage, table scraps, and other forms of food loss and waste for each food item [71]. The Loss-Adjusted Food Availability (LAFA) Data Series was derived from the previous data system [72]. In addition, the ERS uses the above data to estimate food waste and its value at the retail and consumer end of the US, and publishes a report on the evaluation of the food diets of the population through a database to inform the level of nutritional intake [83,84,85].

For the UK, Singer (1979) summarized the results of food loss and waste at the agricultural production, processing, catering, and household levels through research studies [86]. Wenlock et al. (1980) used the bookkeeping method to investigate daily food waste in British households and investigated and analyzed the causes of household food waste with related factors [87]. The UK Waste Recycling Initiative (WRI), on the other hand, is targeting households [88], school canteens [89], and food and beverage services [90] to conduct food waste surveys and issue food waste survey reports.

Adopting a uniform methodology for measuring food waste is an important way for China to systematically address the food waste problem (as shown in Table 1). China can learn from the food waste monitoring mechanisms of Western countries, where direct and indirect measurements are applied in tandem. For the supply and consumption side of the food chain, surveys are conducted according to a range of qualitative and quantitative data to obtain appropriate information. For food waste at the supply side of the food chain, direct measurement can be effective through questionnaires, calculations or scans, diaries, etc., where diaries are regular records of information on the food discarded by individuals or households, etc.

For the food consumption side that can be directly measured, food service entities can make comprehensive calculations through purchase records, sales records, and surplus records, etc. (as shown in Table 2). Household entities can adopt mass balance analysis and coefficient analysis to measure the total amount of all food purchased through purchases, the total amount of food actually consumed, and the total amount of food left over, which are compared with the relevant coefficients.

#### 4.2.3. Improving Food Donation Mechanisms to Prevent Waste at the End of the Supply Chain and Promote the Sharing Economy

Food waste at the end of consumption is very serious in China, with a large amount of quality food being discarded due to a lack of disposal options. The serious mismatch between the supply side and the consumption side both increases the amount of food waste and leads to a significant increase in the cost of food waste disposal. The construction of a food donation mechanism delineates the rights and obligations of redistribution for the participating subjects of food donation, thus forming a unified donation order and promoting the circulation of surplus food. Geneva, Switzerland, launched the “frigo” project, a community refrigerator that allows people to donate and collect food that is about to expire but is still safe to eat, free of charge, thus reducing food waste and contributing to the sharing economy.

China can construct a food donation mechanism. First, the main body of food donation should be clarified and the status and significance of the participating subjects should be determined, such as Italy’s Act No. 166/2016, which stipulates that all actors, donors, and charitable organizations must comply with the General Food Law [92]. Second, a clear definition of donated food should meet all quality standards set by Chinese laws and regulations, even if these products are not sold due to appearance, age, freshness, grade, size, surplus, or other conditions [93]. Finally, a food-sharing platform should be constructed to encourage the movement of food by launching a community sharing program.

#### 4.2.4. Shift in the Regulatory Model from Government Regulation to Multi-Dimensional Co-Regulation

China’s food legislation model is a single regulatory model led by the government. The Anti-Food Waste Law clearly stipulates the rights and obligations of each subject, but due to the large span of the industrial chain and the large differences between different subjects, the government has information asymmetry and market failure in the regulatory process, which leads to poor governance in practice [10]. Although China has organized food conservation campaigns such as the “Empty plate campaign” [94], they have not been translated into regular national action.

China can learn from the 2030 Champions Program and the establishment of ForMat Europe, AECOC, PACTE, and SFA, which combine the theories of social regulation and self-regulation. At the social level, the government sets the rights and obligations for regulated subjects through public social power, which is in line with the position of the theory of the social nature of resources, as corporate entities, unincorporated organizations, etc., on the basis of enjoying social dividends, are obliged to maximize the overall welfare of society. Therefore, administrative means such as tax regulation, administrative penalties, credit evaluation, administrative licenses, administrative coercion, etc. can be carried out and implemented to a limited extent. From an individual point of view, enterprises should add a food compliance component to their compliance system and self-regulate by setting their own standards of conduct to maximize the use of food resources and avoid wastage.

### 4.3. R&D to Drive Innovation in Anti-Food Waste Technology for Sustainable Development

Technology is the first productive force; to promote the effective implementation of an anti-food waste policy, it is necessary to realize the scientific and technological innovations of technology. The EU Circular Economy Package and Farm to Fork Strategy invested a lot of money in food production and recycling technology. Japan, the United States, and the European Union have increased the food cycle and reduced food waste in the production-to-consumption chain by developing technologies to extend the shelf life of food. China can increase its investment in the research and development of food waste technologies to increase the possibility of reducing food waste.

Sustainable development is a comprehensive issue that requires the joint participation of multiple actors. The UK government has signed the Courtauld Commitment with retailers. On the technological level, Too Good To Go develops apps that connect restaurants and consumers, selling leftover food from restaurants to consumers at a discounted price and reducing food waste. Lufthansa introduced artificial intelligence technology to analyze catering trays after flights to understand food waste and catering availability. With the rise of “5G” and artificial intelligence, China can use big data for macro-analysis to balance supply and demand and promote the realization of the Sustainable Development Goals.

Innovations in food waste treatment methods can reduce resource consumption and promote environmental protection from the end of consumption. Current approaches to food waste treatment have taken on scale effects, such as the establishment of food donation systems, composting, anaerobic digestion, and animal feeds, all of which promote sustainable food waste reduction. Innovations in the field of technology will promote the development of new mass productivity, which can be realized through technological innovations, such as biomass energy production and microbial fermentation treatment.

## 5. Conclusions

Addressing the problem of food waste in China is critical to achieving sustainable development and ensuring food security in the face of the growing population pressures and environmental challenges. The Chinese government’s series of efforts have achieved considerable success. The Anti-Food Waste Law has exerted a deterrent effect by clarifying, among other things, who is responsible and how penalties are to be imposed. Catering operators, in particular, have guided consumers to dine sensibly by posting saving signs or suitable menus. However, it remains unique compared to the anti-food waste policies of countries such as the United States, Japan, and the European Union. The introduction of the US 2030 Champions Program made food waste relevant to stakeholders, and the model of collaborative governance fostered practices and innovations to reduce food waste. Japan’s three different periods of anti-food waste strategy shifted from government-led regulation to multi-stakeholder. The European Union promotes sustainable development through a sound food monitoring and regulatory system. China’s unique geography, history, and culture determine the specificity of China’s anti-food waste legislation, but a policy system combining education, technology, culture, and economy and a governance model of pluralistic co-management should be the trend for future reform.

China needs to further clarify its position and strengthen its anti-food waste measures. First, it should improve the accountability mechanism and build a comprehensive governance pattern. Second, it should build a systematic operation mechanism to solve the problem of waste in the food chain in an integrated manner. Thirdly, it should shift from single government regulation to a multi-dimensional model of shared governance, whereby the role of the government in integrating the overall situation and coordinating with all parties should be brought into play. Looking to the future, improving food waste management can lead to enhanced food security by ensuring that more food reaches those in need, thus addressing hunger and malnutrition. Additionally, by promoting sustainability, these initiatives can contribute to broader environmental goals, including reduced greenhouse gas emissions and the conservation of natural resources. Ultimately, the fight against food waste aligns with China’s commitment to sustainable development and its efforts to transition toward a circular economy. China’s fight against food waste is a systematic project, with the ultimate goal of achieving sustainability in food production, transportation, storage, and consumption, which needs to be achieved through the sustainability of systems, policies, technologies, and institutions.

## Figures and Tables

**Table 1:** Waste measurement methods at various stages of the food supply chain [91].

Food Supply Chain Stage	Measurement Methods				
Primary production	Direct measurement	Mass balance	—	Questionnaires and interviews, coefficient and yield statistics, waste composition analysis	
Processing and production					
Retail and other food distribution				Calculate or scan	—
Restaurants and catering services		—	Waste composition analysis		Diary
Household				—	

**Table 2:** Waste measurement methods at various stages of the food consumption chain.

Food Consumption Chain Stage	Measurement Methods		
Catering Services	Incoming goods records	Inputs = Consumption + Exports + Waste + Storage	Equilibrium analysis and coefficient analysis
	Sales records		
	Remaining records		
Household	Total amount of all food purchased		
	Total amount of food actually consumed		
	Total food surplus		

## Data Availability

No new data were created or analyzed in this study. Data sharing is not applicable to this article.
